# Profile of American tegumentary leishmaniasis in transmission areas in the state of Minas Gerais, Brazil, from 2007 to 2017

**DOI:** 10.1186/s12879-020-4860-z

**Published:** 2020-02-22

**Authors:** Mariana Olímpia Köhler Marra Pinto, Tiago Mendonça de Oliveira, Andreza Nayla de Assis Aguiar, Paulo Eustáquio Marra Pinto, David Soeiro Barbosa, Soraia de Araújo Diniz, Marcos Xavier Silva

**Affiliations:** 1 Preventive Veterinary Medicine Department, School of Veterinary, Federal University of Minas Gerais, Belo Horizonte, 31270-901 Brazil; 2 Prefecture of Belo Horizonte, Belo Horizonte, Minas Gerais 31130-908 Brazil; 3 Parasitology Department, Biological Sciences Institute, Federal University of Minas Gerais, Belo Horizonte, 31270-901 Brazil; 4 Centro Universitário UniDoctum de Teófilo Otoni, Veterinary Medicine Course, Teófilo Otoni, Brazil

**Keywords:** Cutaneous leishmaniasis, Epidemiology, Public health, Zoonoses

## Abstract

**Background:**

American tegumentary leishmaniasis (ATL) is a widespread anthropozoonosis caused by protozoa of the genus *Leishmania* and is considered a serious public health problem. The aim of this study was to provide a descriptive analysis of confirmed ATL cases and evaluate the spatial distribution of ATL in high-risk transmission areas from the state of Minas Gerais, Brazil.

**Methods:**

An ecological, analytical, and retrospective study of the confirmed cases of ATL in Minas Gerais from 2007 to 2017 was conducted. To characterize these cases, multiple correspondence analysis and georeferencing of the ATL prevalence rates in the municipalities were conducted based on variables obtained at Sistema Nacional de Agravos de Notificação and Instituto Brasileiro de Geografia e Estatística databases.

**Results:**

There were 13,025 confirmed cases of ATL from 74.4% (635) municipalities of Minas Gerais, corresponding to a prevalence rate of 66.5 cases for every 100,000 inhabitants. Males aged 20 to 59 years and individuals who attended elementary school were most affected with ATL. Multiple correspondence analysis presented an accumulated qui-squared value of 44.74%, proving that there was a relationship between the variables, including ethnicity, age, pregnancy status, zone of infection, and number of cases.

**Conclusion:**

We confirmed that ATL is endemic to Minas Gerais, and there is high risk of infection within the municipalities due to a high rate of parasite transmission. The occurrence of infection in children, pregnant women, and the indigenous population demonstrates the need for the government to expand social policies aimed at vulnerable groups.

## Background

American tegumentary leishmaniasis (ATL) is a widespread zoonotic disease that has a global impact [1]. It is caused by the protozoa of the genus *Leishmania* and is considered a serious public health problem [2]. Phlebotomine sand flies, such as *Nyssomyia* spp*.* and *Lutzomyia* spp., belonging to the family Psychodidae (subfamily Phlebotominae), act as vectors for *Leishmania* in the Americas and play an important role in disease dissemination among humans and domestic animal reservoirs [3–5]. In Brazil and South America, the major agent causing ATL is the *Leishmania (Viannia) braziliensis* [6].

Recently, ATL has been reported in all Brazilian states, and its epidemiological pattern has gone through changes regarding its transmission, with the occurrence of peaks every five years [7]. From 2007 to 2017, 232,989 cases of ATL were reported in Brazil, with a mean prevalence of 118.39 cases annually for every 100,000 inhabitants.

In the state of Minas Gerais, ATL was first reported in 1940, and its transmission has been reported from both rural and peri-urban areas [8]. Thus, the objective of the present study was to use descriptive analysis to characterize ATL cases from the state of Minas Gerais and demonstrate their spatial and temporal distribution from 2007 to 2017.

## Methods

### Development of the study

An ecological, analytical, and retrospective study of confirmed cases of ATL, which occurred between 2007 and 2017 in Minas Gerais, was conducted using the secondary database established by the *Sistema de Informação de Agravos de Notificação* (SINAN) and the Brazilian Institute of Geography and Statistics, also known as *Instituto Brasileiro de Geografia e Estatística* (IBGE).

### Location of the study

The state of Minas Gerais is located in the southeastern region of Brazil and is divided into 853 municipalities. Its territorial area is 586,520.732 km^2^, and its vegetation is composed of 19.94% savannah, 10.33% Atlantic forest, and 3.48% caatinga; 33.8% of the total area is native forest [9, 10]. According to the IBGE census conducted in 2010, Minas Gerais had a population of 19,597,330 inhabitants, corresponding to a population density of 33.41 inhabitants per km^2^ [9].

### Database

A total of 635 municipalities in Minas Gerais, which reported confirmed cases of ATL were considered as analytical units, and epidemiological aspects that could characterize these cases were evaluated using data obtained from SINAN. Demographic variables, such as gender, age, education level, zone of infection, and ethnicity were evaluated, in addition to variables, such as pregnancy status, annual and monthly frequency of disease, and ATL confirmation criteria. These variables were dichotomized or categorized to perform a multiple correspondence analysis. An adaptation of the criteria presented by [7, 11] was used to classify the epidemiological condition of ATL cases from Minas Gerais, which considered the prevalence rate of each municipality positive for ATL. The number of ATL cases within each municipality was categorized as “low” for 1–20 cases, “moderate” for 21–50 cases, and “high” for 51–828 cases.

### Statistical analyses

Multiple correspondence analysis of the data was performed using the software Stata 12.0 (Stata Statistical Software: Release 12. College Station, TX: StataCorp LP). Variables that comprised the correspondence model were selected using Pearson’s chi-square test (*P* ≤ 0.05) and evaluated for possible associations between the number of cases and other variables using graphs, which were interpreted by evaluating the proximity of the variables under the category “number of cases.” The intensity of association with values of cumulative inertia above 40% was considered [12].

For the epidemiological analysis of ATL from 2007 to 2017 in Minas Gerais, a control diagram was designed by calculating the mean, upper and lower limits, and standard deviation. By using this diagram, it was possible to evaluate the temporal evolution of the disease and detect possible alterations in its distribution pattern. The diagram data can be used to developing effective protective measures against the ATL [13].

### Georeferencing

**Fig. 1:**
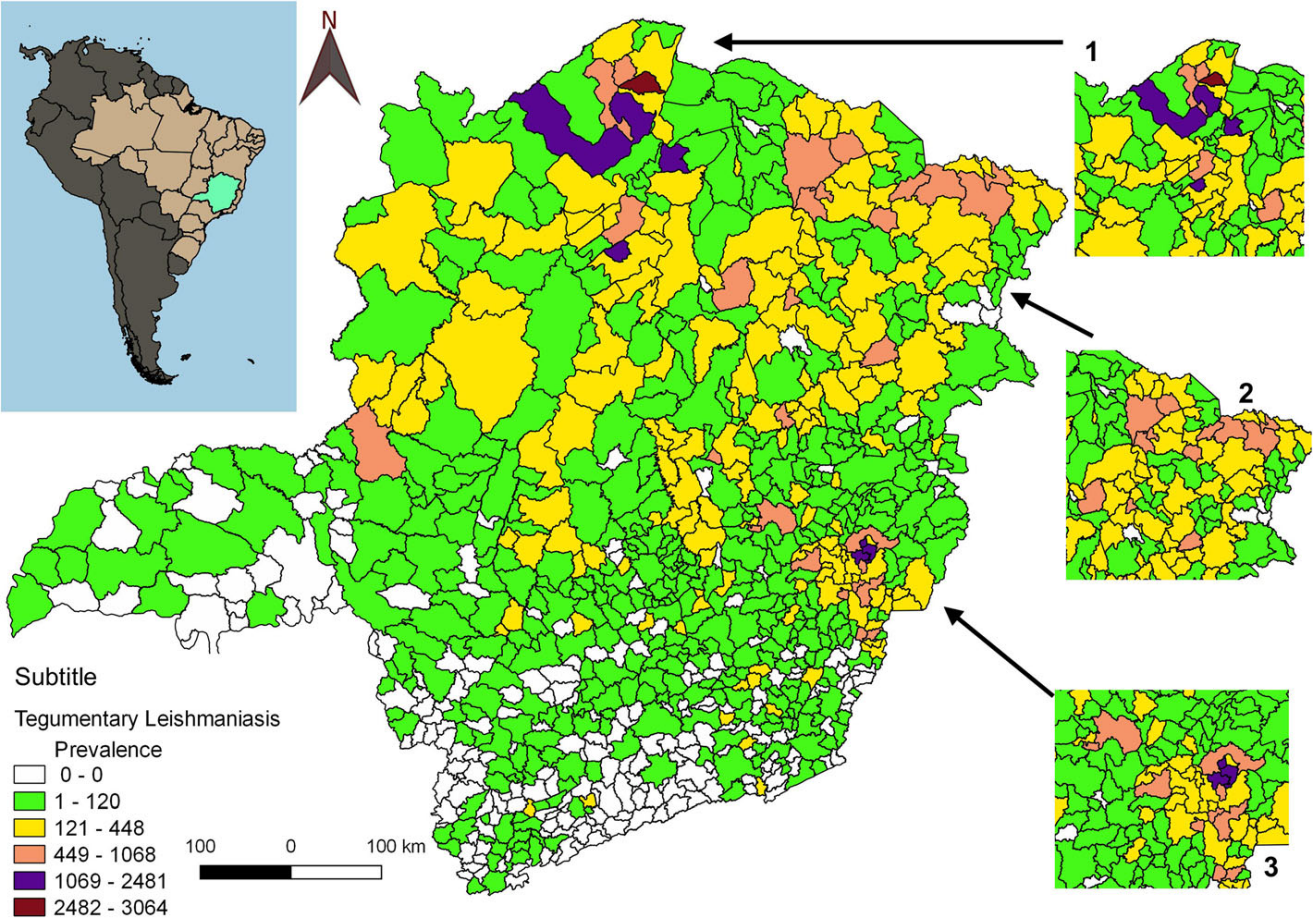
Distribution of the ATL confirmed cases in Minas Gerais, Brazil from 2007 to 2017

The prevalence rate of ATL in the municipalities, was calculated in a 95% confidence interval based on data obtained from SINAN, and a georeferencing map was constructed. The software QGIS 2.18.14 (QGIS Development Team, 2009, QGIS Geographic System, Open Source Geospatial Foundation) was used to map the spatial distribution of municipalities with prevalent infection using data for confirmed cases of ATL that occurred in Minas Gerais from 2007 to 2017 (Fig. 1). After the calculation of the prevalence rate, 20 municipalities were identified as having prevalent infection, with the highest number of cases observed in the state of Minas Gerais (Table 1).

**Table 1:** Cases of American tegumentary leishmaniasis (ATL) in areas with a high risk of transmission in Minas Gerais (2007 to 2017)

Municipalities with reported cases	No. of cases	Population (2010)	Prevalence rate*
São João das Missões	359	11,715	3064.4
São João do Pacuí	98	4066	2410.2
São Domingos das Dores	86	5396	1593.8
Varzelândia	273	19,126	1427.4
Januária	828	65,464	1264.8
Ubaporanga	138	12,040	1146.2
Imbé de Minas	69	6412	1076.1
Simonésia	187	18,302	1021.7
Piedade de Caratinga	66	7101	929.4
Cachoeira de Pajeú	68	8962	758.8
Novorizonte	37	4953	747.0
Vermelho Novo	35	4689	746.4
Rio Pardo de Minas	216	29,075	742.9
Inhapim	170	24,269	700.5
Cônego Marinho	47	7089	663.0
Teófilo Otoni	218	134,733	161.8
Montes Claros	514	361,971	142.0
Ipatinga	257	239,177	107.5
Patos de Minas	147	138,836	105.9
Belo Horizonte	221	2,375,444	9.3

Subtitle: * per 100,000 inhabitants; Minas Gerais presented a prevalence rate of 66.5 cases for every 100,000 inhabitants

## Results

From 2007 to 2017, 13,025 confirmed cases of ATL from 74.4% (635) municipalities of Minas Gerais (Fig. 1) were reported, corresponding to a prevalence rate of 66.5 cases for every 100,000 inhabitants. One third of these cases were reported from 15 municipalities evaluated in this study (Table 1). The municipalities of São João das Missões, São João do Pacuí, São Domingos das Dores, Varzelândia, and Januária had the highest prevalence rates of ATL. Data for Belo Horizonte, Teófilo Otoni, Montes Claros, Ipatinga, and Patos de Minas were included in the variable analysis owing to their importance as economic centers of the state and because they reported a moderate to high frequency of the disease.

A total of 90.65% cases were diagnosed using clinical-laboratory criteria, while 9.35% of cases were diagnosed using clinical-epidemiological criteria. Laboratory diagnosis involved immunological examinations, such as the Montenegro intradermoreaction, parasitological tests with direct visualization of the parasite, in vitro isolation and culture, in vivo isolation, and polymerase chain reaction. The clinical-epidemiological criteria for diagnosing cutaneous leishmaniasis were applied only to individuals without access to laboratory diagnostic services and with residence, origin, or displacement to an area with confirmed cases.

When considering gender and age variables, male individuals (60.21%) and adults aged 20 to 59 years (56.89%) were seen to be the most affected. The education-level variable showed that approximately 36% of the cases reported in the highlighted municipalities, were those of individuals who had elementary schooling. However, 50% of notification forms did not include this information in these locations. Upon evaluating the role of ethnicity in disease prevalence, we verified that the individuals most affected by ATL were brown skinned (44.05%). There were 24 cases of pregnant women with ATL, representing 0.59% of all forms of ATL notifications in high transmission areas (Table 2).

**Table 2:** American tegumentary leishmaniasis case description related to epidemiological and demographic variables from 2007 to 2017, Minas Gerais, Brazil

Variable	n (MG scenario)	%	n (municipalities with highest prevalence rates*)	%
Gender				
Male	7926	60.85	2429	60.21
Female	5097	39.14	1605	39.79
Ignored	2	0.01	_	_
Ethnicity				
White	4174	32.05	1024	25.38
Black	1210	9.29	192	4.76
Yellow	134	1.03	32	0.79
Brown	5827	44.74	1777	44.05
Indigenous	369	2.84	341	8.45
Ignored	1311	10.05	679	16.83
Age range				
Child (younger than 1-year-old)	143	1.09	33	0.82
Child (1–14 years old)	1817	13.95	730	18.10
Teenager (15–19 years old)	902	6.93	361	8.95
Adult (20–59 years old)	7579	58.19	2295	56.89
Elderly (60 years old or older)	2584	19.84	615	15.25
Education level				
Illiterate	705	5.41	138	3.42
Elementary school degree	5685	43.65	1441	35.72
High school degree	1293	9.93	319	7.91
Graduation degree	312	2.39	109	2.70
Ignored / Not applied	5030	38.62	2027	50.25
Pregnancy				
Not applied	12,518	96.11	3836	95.09
Yes	79	0.60	24	0.59
Ignored	428	3.29	174	4.31
Confirmation criteria				
Clinical-laboratory	11,023	84.63	3657	90.65
Clinical-epidemiological	2002	15.37	377	9.35
TOTAL	13,025	100.00	4034	100.00

Subtitle: (*) every 100,000 inhabitants

Ethnicity data is based on the IBGE demographic census, as shown on the website <https://educa.ibge.gov.br/jovens/conheca-o brasil/populacao/18319-cor-ou-raca.html>

The prevalence of confirmed cases of ATL is highlighted in Fig. 1. A cluster can be observed in north Minas Gerais, Rio Doce (east), and Jequitinhonha valley. The southern and Mineiro Triangle regions presented low to moderate prevalence.

**Fig. 2:**
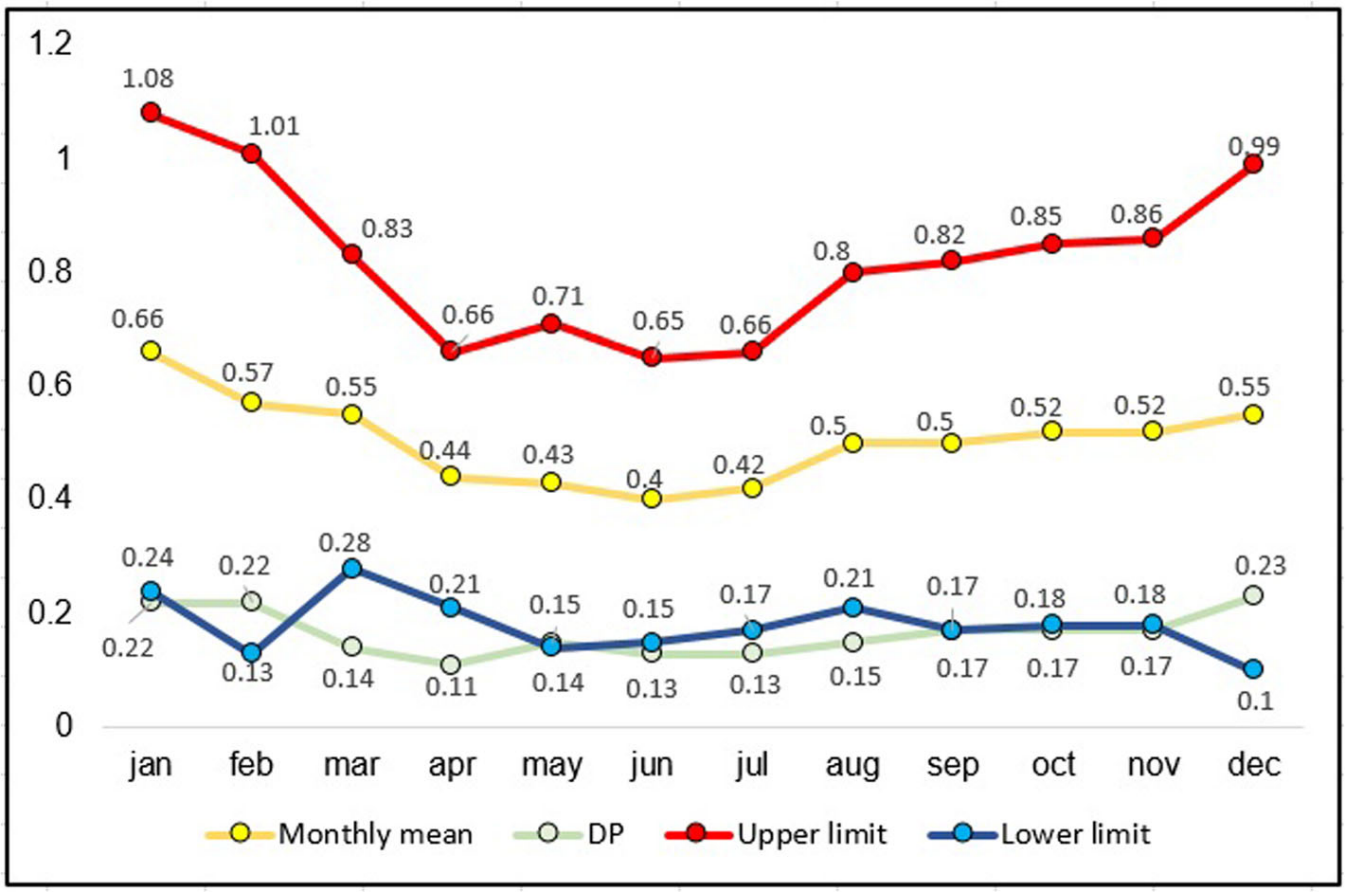
Control diagram according to the monthly average frequency of ATL cases in Minas Gerais, from 2007 to 2017

The control diagram describes the expected ATL case average in Minas Gerais from 2007 to 2017 and suggests that Minas Gerais is an endemic location for the disease. Moreover, the average number of cases was slightly higher in the months when the temperature was warmer compared to the average number of cases during the colder months (Fig. 2).

**Fig. 3:**
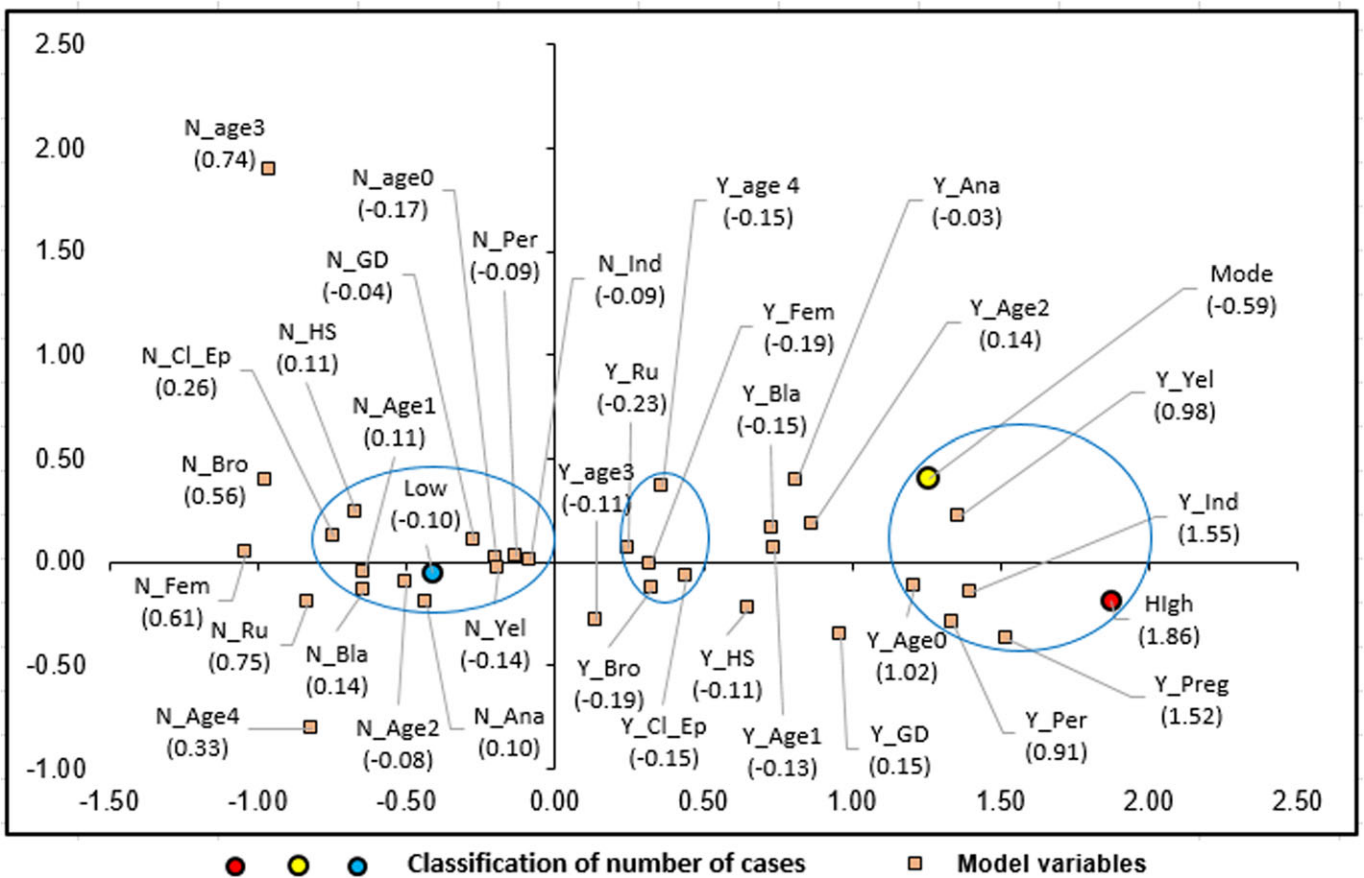
Graphic of the Multiple Correspondence Analyses of the epidemiological of the ATL cases occurred between 2007 and 2017 in Minas Gerais, Brazil. Low: municipalities with low number of cases; Mode: municipalities with moderate number of cases; High: municipalities with high number of cases; N_Ana: not illiterate; Y_Ana: illiterate; N_HS: do not have high school degree; Y_HS: have high school degree; N_GD: do not have graduate degree; Y_GD: have graduate degree; N_Preg: not pregnant; Y_Preg: pregnant; N_Age0: not age 0 (younger than 1-year-old); Y_Age0: age 0 (younger than 1-year-old); N_Age1: not age 1 (1–14 year-old); Y_Age1: age 1 (1–14 years old); N_Age2: not age 2 (15–19 years old); Y_Age2: age 2 (15–19 years old); N_Age3: not age 3 (20–59 years old); Y_Age3: age 3 (20–59 years old); N_Age4: not age 4 (60 years old or older); Y_Age4: age 4 (60 years old or older); N_Cl_ep: not evaluated using clinical-epidemiological criteria; Y_Cl_ep: evaluated using clinical-epidemiological criteria; N_Bla: not black patients; Y_Bla: black patients; N_Yel: not yellow patients; Y_Yel: yellow patients; N_Bro: not brown patients; Y_Bro: brown patients; N_Ind: not Indian patients; Y_Ind: Indian patients; N_Fem: not female patients; Y_Fem: female patients; N_Per: not peri urban area; Y_Per: peri urban area; N_Ru: not rural area; Y_Ru: rural area

The correspondence analysis plot, demonstrating the epidemiologic profile of ATL in Minas Gerais, presented an accumulated chi-squared value of 44.74%, and the evaluated variables of the model have been highlighted by blue circles in Fig. 3. We observed that the number of case variables corresponded with variables, such as age, ethnicity, and education level, zone of infection, and pregnancy status. The associations among a set of variables, such as age, gender, ethnicity, zone of infection, and confirmation criteria was also verified.

We observed that municipalities with a moderate (21 to 50) to high (51 to 828) number of confirmed ATL cases were associated with the following characteristics: pregnancy, indigenous population, peri-urban zone, yellow-skinned individuals, and children younger than 1-year-old. Municipalities with a low number of ATL cases (1–20) were associated with characteristics, such as education level, age range, skin color (not black- or yellow- skinned), and non-indigenous origin. The confirmation criteria were not clinical and epidemiological, and the infection did not occur in peri-urban areas.

An association among brown skin, age 4 (60 years or older), female sex, infection in rural areas, and diagnosis made based on clinical-epidemiological criteria was also analyzed.

## Discussion

In the last 11 years, 13,025 cases of ATL were confirmed in Minas Gerais, and the highest prevalence rate was observed in municipalities from the north of the state and from Rio Doce (east) and Jequitinhonha Valley. These regions are known as zones of low development and poverty and present significant diversity among the phlebotomine species. These features may be related to the dissemination of ATL to humans and domestic animals, which act as reservoirs, in these areas. In addition, anthropogenic factors are also determinants of disease maintenance [14–16].

We verified that the most effective confirmation criteria were those from the clinic and the laboratory. Confirmation of ATL using the clinical-epidemiological criteria provides important epidemiological information, as it identifies the parasite and may help in taking control measures against it. This result is in agreement with the Ministry of Health Guidance for Health Surveillance (2017) [7], which claims that the parasitological method for ATL diagnosis may be conducted before the onset of treatment, especially in cases where the clinical evolution is not ordinary and/or there is a bad response to treatment.

Analysis of data on the age and gender of patients with ATL in Minas Gerais revealed that males of working age were more susceptible to the disease, a finding that corroborates with those of previous reports [17, 18]. This might be because the occupations of males in this region, such as military occupation and construction, put them at a higher risk of being exposed to ATL vectors [19, 20].

The low education level among most patients with ATL might be associated with a poor understanding of risk factors related to the disease. The occurrence of ignored notification forms this variable (50.25%) might make it difficult to implement educational measures targeted to specific risk groups according to the degree of education. Additionally, the absence of educational level data makes it impossible to evaluate this criterion and is a limitation of this study.

Concerning the ethnicity of the patient, the ATL infection pattern agrees with the racial-ethnic characteristics of the Brazilian population demonstrated by IBGE (2010) [11]. According to IBGE, there is a larger brown skinned population in Brazil compared to the populations of other ethnicities. However, São João das Missões, a municipality located 663 km from the capital Belo Horizonte also has a strong presence of indigenous populations. There are a total of 33 villages occupied by the Xakriabá in Belo Horizonte [21]. In these villages, 359 cases of ATL were reported over a period of 11 years; 333 (92.8%) occurred within the indigenous population. Moreover, this municipality presented the highest prevalence of ATL in Minas Gerais during the study period (3064.4 cases per 100,000 inhabitants). Thus, it is considered an area of high risk for the transmission of *Leishmania* (Fig. 1 and Fig. 3). The data demonstrated greater susceptibility to *Leishmania* infection within the indigenous population. Furthermore, a previous study [22] had suggested that the high prevalence of ATL in this area was due to the presence of a large number of wild reservoirs and high rates of deforestation. The main species involved in this area was *L. braziliensis*, in agreement with data presented by a study conducted in 2017 [22, 23], which reported on the presence of *L. braziliensis* in individuals from an indigenous village in Mato Grosso.

ATL infection consequences for pregnant women and maternal-fetal health are rarely and poorly studied. In the present study, pregnant women represented 0.59% of all ATL cases in Minas Gerais. This result agrees with those of previous findings [24, 25], which identified only 27 pregnant women with ATL among a group of 4200 people with the disease. Morgan et al. [24] reported pregnancy-related complications in patients who delivered preterm or had stillbirth, with a frequency of 10.5% for each group. The municipality of Januária, located 559 km from the capital city, was considered as a high transmission site in this study, presenting 11 cases (13.9% of a total of 79) of pregnant women diagnosed with ATL and, thus, becoming the main focus for analyzing this variable in Minas Gerais (Fig. 2).

The MCA results showed that the peri-urban zone of infection was related to the municipalities that presented moderate to a high number of ATL cases. These results agree with those described in the manual of ATL surveillance [7] and with those of the survey conducted in 2017 [26], which considered the peri-urban zone an important infection area for the disease. Moreover, the literature suggests that the adaptation of the phlebotomine subfamily and anthropic activities that impact the environment are responsible for ATL transmission in the peri-domestic environment. For example, breeding animals close to households favored vector adaptation to the human domain and modified the ATL transmission cycle [27].

In the correspondence analysis, some associations between disease in pregnant women and children younger than 1-year-old and indigenous/yellow ethnicity was found. This might explain the higher susceptibility of these populations to ATL and can be linked to characteristics related to exposure to the vector and vector adaptation to the peri and/or intra-domestic areas [28].

The results observed for the associations among brown skin, age 4 (60 years or older), females, infection in rural areas, and disease confirmation using the clinical-epidemiological criteria are similar to those obtained in other published reports [2]. Furthermore, the present study verified that adult males are the most affected, while children and women are affected to a lesser degree.

Analysis of the control diagram showed that ATL is endemic to Minas Gerais, as the data on disease frequency in this area are similar to the results seen in the past in the absence of epidemics. In addition, we verified that during cold months there was a slight decrease in the occurrence of ATL cases, which demonstrated a correlation between temperature and phlebotomine activity, as described in a study conducted in 2017, which compared the activity of the vector during different seasons [29].

When taking the entire country into account, the average prevalence rate for ATL was approximately 118.9 cases for every 100,000 inhabitants, suggesting that the situation in Minas Gerais was less critical, as it has an average prevalence rate of 66.5 cases for every 100,000 inhabitants. However, the cutoff that determined whether certain locations were at a high risk, is 448.3 cases for every 100,000 inhabitants [11]. In this study, we found that the municipalities highlighted in Table 1 are at a higher risk of leishmaniasis transmission.

## Conclusion

The present study provides a new perspective on ATL as it involved a multivariate analysis using multiple correspondence analysis and used epidemiological and demographic variable data openly available on SINAN and IBGE.

We verified that ATL was endemic to Minas Gerais, especially in municipalities with high transmission rates. The occurrence of the infection in children, pregnant women, and indigenous populations demonstrates the need for the government to implement social policies directed towards these vulnerable groups. Thus, it is essential to fully understand the epidemiology of ATL to implement control measures against this parasite.

ATL is a health priority in certain municipalities of Minas Gerais. Our study evaluated disease epidemiology and discussed the need for public policies to address ATL. Recently, Brazil decentralized its health services; such changes may worsen disease incidence and prevalence in this country, as the country has been struggling economically and lacks human and material resources required for disease control.

Cases of ATL notified through a secondary database can be a limitation for this type of epidemiological study as they can lead to an underestimation of disease prevalence, due to under-reporting, and cause collection bias. Moreover, ecological studies can be susceptible to ecological bias as they do not allow for analysis of individual data.

## Data Availability

All data presented in the study can be accessed in the SINAN and IBGE databases (available at http://datasus.saude.gov.br and http://www.ibge.gov.br, respectively).

## Abbreviations

High: Municipalities with high number of cases
Low: Municipalities with low number of cases
Mode: Municipalities with moderate number of cases
N_Age0: Not age 0 (younger than 1-year-old)
N_Age1: Not age 1 (1–14 year-old)
N_Age2: Not age 2 (15–19 years old)
N_Age3: Not age 3 (20–59 years old)
N_Age4: Not age 4 (60 years old or older)
N_Ana: Not illiterate
N_Bla: Not black patients
N_Bro: Not brown patients
N_Cl_ep: Not evaluated using clinical-epidemiological criteria
N_Fem: Not female patients
N_GD: Do not have graduate degree
N_HS: Do not have high school degree
N_Ind: Not Indian patients
N_Per: Not peri urban area
N_Preg: Not pregnant
N_Ru: Not rural area
N_Yel: Not yellow patients
Y_Age0: Age 0 (younger than 1-year-old)
Y_Age1: Age 1 (1–14 years old)
Y_Age2: Age 2 (15–19 years old)
Y_Age3: Age 3 (20–59 years old)
Y_Age4: Age 4 (60 years old or older)
Y_Ana: Illiterate
Y_Bla: Black patients
Y_Bro: Brown patients
Y_Cl_ep: Evaluated using clinical-epidemiological criteria
Y_Fem: Female patients
Y_GD: Have graduate degree
Y_HS: Have high school degree
Y_Ind: Indian patients
Y_Per: Peri urban area
Y_Preg: Pregnant
Y_Ru: Rural area
Y_Yel: Yellow patients
